# Network motifs for translator stylometry identification

**DOI:** 10.1371/journal.pone.0211809

**Published:** 2019-02-08

**Authors:** Heba El-Fiqi, Eleni Petraki, Hussein A. Abbass

**Affiliations:** 1 School of Engineering and Information Technology, University of New South Wales, Canberra, ACT, Australia; 2 Faculty of Education, University of Canberra, Canberra, ACT, Australia; University of Sao Paulo, BRAZIL

## Abstract

Despite the extensive literature investigating stylometry analysis in authorship attribution research, translator stylometry is an understudied research area. The identification of translator stylometry contributes to many fields including education, intellectual property rights and forensic linguistics. In a two stage process, this paper first evaluates the use of existing lexical measures for the translator stylometry problem. Similar to previous research we found that using vocabulary richness in its traditional form as it has been used in the literature could not identify translator stylometry. This encouraged us to design an approach with the aim of identifying the distinctive patterns of a translator by employing network-motifs. Networks motifs are small sub-graphs which aim at capturing the local structure of a complex network. The proposed approach achieved an average accuracy of 83% in three-way classification. These results demonstrate that classic tools based on lexical features can be used for identifying translator stylometry if they get augmented with appropriate non-parametric scaling. Moreover, the use of complex network analysis and network motifs mining provided made it possible to design features that can solve translator stylometry analysis problems.

## Introduction and motivation

A much-debated question about translation is whether the translation is an art, science, or art and science combined. This question is raised due to the very specific nature of the translation task. If a piece of text is being given to two translators to translate, how can their correctness, validity, and accuracy be measured? What causes people to prefer one of these translations over another? Do translators have their own touch or signature in their translations? Or is it the case that if we have a number of valid translations for the same text, all of them are indistinguishable?

Authors use words to communicate the mental pictures in their mind to their readers. If we have different translations for a piece of text that a particular author wrote, the best translation is the one that is able to deliver the closest mental picture that the author drew in the original text. In order to do that, translators do not only map words from one language to another, but also they have to make many decisions to deliver the meanings, feelings, rhythms, tone, and diction of the original author. Therefore, many scholars treat the translation work as a combination of art and science [1, 2]. That in turn led us to understand the existence of a translator style as a form of art which is the position taken in this paper. Stylometry analysis is the field of research that investigates how writing patterns may vary from one writer to another. It is a well-established research area that has benefited from the development of powerful computational tools in the last decades. However, the translator stylometry problem is one of the under-researched areas within the computational linguistics perspective. There have been many literary studies discussing how parallel translations to the same language carried out by different translators may vary in the delivery of meanings, feelings, and character representations to the readers [3–9]. However, the number of research studies that used computational linguistics for translator stylometry is very limited.

One reason for this phenomenon is due to the extra challenges associated with this problem where translators are always asked to be invisible in their writing in addition to the limited freedom of expression that the translators have while translating. Another reason for the limited research conducted in this area is the output and conclusions of the existing studies that failed to identify translator stylometry computationally [10, 11]. Mikhailov and Villikka’s study suggested that translator stylometry cannot be detected using computational linguistics [10]. Hedegaard and Simonsen originally considered the translator effect in the text as noise that challenged identifying the author of the text rather than considering the translator’s intellectual contribution to the work [12]. In a more recent study, Rybicki [11], supporterd Mikhailov and Villikka’s claim when he questioned the translator stylometry identification using clustering analysis and he found the translations to be grouped based on their original authors rather than their translators. Our research, including this paper, address this challenge.

Despite the limited research on the topic, identification of translator stylometry is an important topic and has different areas of application. These include detecting plagiarism in translation classes, addressing differences between expert and learners, or resolving intellectual property cases in the legal domain.

Translator intellectual property is a contentious topic due to market practices of considering the translation a simple task rather than an art that has its intellectual property. The most famous dispute case is regards to the popular novel “The lord of the Rings”. This novel had been translated by Lenita Esteves in the 90’s before the release of the movie that turned this book into a bestseller book. Esteves was surprised that the subtitles in the Brazilian version of the movie had been taken from her translation including the character’s names and some poem lines as well without her permission. Therefore, she sued both of the publishing house and the movie distributor. The publishing house rejected her claim based on what they called the current “market practice”. They argued that this was the practice at the time whereby translators were not paid for copyright, but only for the task of translation. We hope to contribute to this debate, by demonstrating the existence of translator styles computationally.

In this research study, we aim at answering the following research question: “Can a computational linguistics framework address the challenges associated with the translator stylometry identification problem?”

In order to answer this question, we will simplify this question toward a specific scenario where we will be given a parallel translation of the same original text (translation_1_ and translation_2_) that belongs to two translators (A and B). Then, we will test if the computational linguistic framework that we offer is able to map these translations to their actual translator or not.

The following section presents a brief literature on translator stylometry problem in both linguistics and computational linguistics studies. Section 3 justifies the choice and design of the corpus used in this study. Section 4 describes the newly proposed method. Section 5 discusses the experimental design and presents the results with their analysis. Finally, the last section concludes with a reflection on the initial problem of translator stylometry and the use of network analysis for identifying translator stylometry followed by suggestions for future research in the field.

## Background

*Stylometry* is the study of the unique linguistic styles and writing behaviours of individuals. Kestemont defined it as the quantitative study of (literary) style, nowadays often accomplished by means of computation [13]. Stylometry can be thought as a measure of the style of a writer, which begs the question of what a style is.

“*Style* is the variable element of human behaviour” [14]. Typical human activities carry invariant similarities. How people get dressed, eat, or drive are generally invariant, but also they slightly vary from one person to another. The general stpdf followed in these activities are invariant among people. Nevertheless, there are salient differences in the parameterisations of these stpdf and choices made along the way for fine grained implementations. These choices will vary noticeably from one person to another.

Style in written language is generated by the repeated choices that the writer tends to make, sometimes subconsciously. These repeated choices are hypothesised to reflect an author’s preference of some writing patterns over others. Group and individual variations in written language can be manifested in the examination of style.

Linguistic group variations have been observed and researched in sociolinguistics and discourse studies; a field that examines the patterns of language use in specific contexts and/or the effect of social factors such as age, gender, and ethnicity on language choices [15, 16].

There are many aspects that may influence language use. Examples of these aspects include people’s background, their level of education, gender, ethnic group, and profession. For example, a sociolinguistic study by Argamon et.al showed that males tend to use determiners (a, the, that, these) and quantifiers (one, two, more, some) more than females. On the other hand, females use pronouns (I, you, she, her, their, myself, yourself, herself) more frequently than males [17].

While social variations are common, there are also individual variations specific to individuals that constitute their own unique personal writing styles. With respect to writing, individual variations are created by the writer’s decision to choose one particular form out of the assortment of all different possible forms. These variations can be within the norm. They are different ‘correct’ ways of expressing the same thing, or they can be deviations from a norm such as mistakes, or idiosyncratic behaviours of the writers. McMenamin [14] offers an example to describe grammatically correct variations within the norm: if the norm is “I am going now”, a variation within the norm could be “I’m going now”, and a deviation could be “I be goin’ now”.

Another example which describes a socially appropriate variation to the norm “I’m afraid you’re too late” is “Sorry, the shop is closed”. In this case, a deviation may be “Get the hell out of here!”. As the style constitutes distinctiveness, identifying a writer’s distinctive markers is the key to identifying her style. Analysis of the variation is the first step towards identification of style-markers.

Current authorship attributions’ features and methods in the literature are not well evaluated against adversarial stylometry [18]. Privacy research drove the initial work in adversarial stylometry. Brennan et al. demonstrated that certain linguistic features failed under adversarial conditions [19]. In that study, evaluation of obfuscation and imitation by nonexperts were evaluated in addition to two-step machine translations. In two-step machine translation, the first step is carried out on the lexical level with some reordering to match the syntax of the target language, and second conjugation and declination is carried out in the target language. The findings from the study demonstrate the weaknesses of existing stylometry features for authorship attribution; demonstrating the need for examining the robustness of stylometric features against manipulation before these features qualify as evidences in a court.

### Translators stylometric analysis (problem definitions)

The way of handling and discussing the translator’s stylometric analysis in the literature has varied according to the purpose of the study. Researchers interested in investigating the causes behind the variations in the styles produced by different translators focused on the literary side of the analysis. On the other hand, researchers who focused on identifying measurable features of stylometric identification focused on the computational linguistic side of the analysis. Therefore, the translator stylometric analysis problem can be divided into two sub-problems: the first is Translator profiling and the second is Translator stylometry identification. Both sub-problems are discussed below.

#### Translator profiling

The background of a translator has been shown to affect the translators’ style. Most of the studies analysing translation in the literature targeted this area of research. Researchers were interested in analysing how two translations of the same text by two different translators (which we denote as parallel translations) differed in delivering different meanings and mental pictures based on the identity of their translators. This includes their cultural [20], social [20], gender [21, 22], and proficiency level [8] backgrounds. Most of the research in this area analysed two different parallel translations originating from the same text, that were translted by two different translators, to address how their identities might have affected the choices they made throughout the process of text translation. Researchers used different linguistic approaches to detect translator styles.

A translator’s style has been described as being beyond the translator’s cognition using Relevance theory [23]. In her study, Xiumei revealed that the decisions a translator makes while trying to communicate the author’s intended message to the reader’s cognitive environment, is affected by her own identity as an individual and this happens unconsciously [23].

Rybicki used Burrow’s Delta to investigate character idiolects in two English translations of Henryk Sienkiewicz’s Trilogy in terms of major characters, old friends, nationality, characters in love, and idiolects of female characters. That study found that character’s idiolects were preserved in translations. Burrow’s Delta was able to capture similar distances between characters in both the original text and the translations [24].

Explicitation happens when the translator transfers a message that was hidden (but can be understood from the context) in the original text to the reader explicitly using the target language. Implicitation, on the other hand, occurs when the translator uses the target language to conceal some details that were mentioned explicitly using the source language. Explicitation effect on translator’s style has been investigated by Kamenická using two parallel English to Czech translations of “Small World” by David Lodge and “Falconer” by John Cheever in 2008 [3]. Kamenická findings conclude that the two translators use experiential and interpersonal explicitation and implicitation in textual segments differently.

Some research studies investigated gender identification of a translator. In 2007, Leonardi conducted a contrastive analysis of an Italian to English translation corpus to address the question of how gender and ideology affect translation [21]. The same question was addressed again in 2011 by Sabet and Rabeie [22]. They studied the effect of a translator’s gender ideology on translation using two Persian translations of the English Novel “Wuthering Heights” by Emily Brontë, one of them by a male translator and the other by a female translator.

In 2009, Castagnoli investigated the possibility of a relationship between the occurrence of specific phenomena and translator competence [8]. She used a corpus consisting of student translations (from English to Italian) and (from French to Italian). That corpus provides the availability of multiple parallel translations of the same original text and availability of different levels of translation competency.

Winters conducted multiple studies on how a translator’s attitude influences his/her translation [4–7]. In all of these studies, Winters used two German translations of the original novel “The Beautiful and Damned” (1922) written by F. Scott Fitzgerald.

In 2004, Winters used loan words and code switches to differentiate between translators’styles [4]. The analysis showed that one of the translators tended to transfer English words from the source text into the translation where possible, while the other translator tended to Germanize the words to transfer the source text culture for the target language reader.

Later on, in 2007, Winters used speech-act report verbs [5] to investigate their usefulness as potential elements of a translator’s individual style. Although the original text used repetition of some words, one of the translators transferred that repetition to the translation, but the other translator avoided that and used different words to reflect different situations.

In a 2009 study, Winters conducted a quantitative analysis to analyse the use of modal particles by the translators. That research showed that despite the overall similarities in using modal particles, there was a significant difference in the translator’s choices and uses of individual modal particles [6]. In 2010, Winters’study showed that different translators’ views affect the macro level of the novel, in which, the main message delivered by the translations of the novel is different. The focus of one translator was to provide a character study while the other focused on societal issues. Furthermore, Winters discussed how that may extend to influence the readers’ attitude as well [7].

Li et.al [20] tried to capture differences in the translation styles of two English translations of a classic Chinese novel “Hongloumeng”. They calculated Type/token ratios, sentence length, and vocabulary. The analysis in that study aimed at differentiating between the translator styles based on the social, political, and ideological contexts of the translations. They also explored the effect of the translator’s native language on their translation style as one of the translators was a Chinese native speaker, and the other was a British scholar. They found that the two translators used two different strategies in translation. The contribution of this study was that variations that have been found between the two translations were caused by their social, political, ideological preferences, as well as their primary purpose of the translations.

A list of keywords was also used for translators’ fingerprints identification by Wang and Li in two parallel Chinese translations of Ulysses. They identified translator preferences for specific keywords. They also found differences on the syntactic level by analysing the decision of clause positions in the sentences [9]. Additionally, their findings affirmed a hypothesis that they made in their study that a writer’s preferences of linguistic expression are demonstrated in free writing.

All the above studies offer sufficient evidence for the existence of translators’ fingerprints in their translations. They also provide substantial background for this research. From the above review we can conclude that there has been wide variations in the approaches used to study translator stylometry; however, none of these research studies employed data mining and machine learning, even in the quantitative oriented studies. This presented a strong motive to conduct this research.

#### Translator identification

These aforementioned studies on translation identification revealed how translators use linguistic features differently to deliver the same original text. Their identities are reflected in the choices that they make while translating. Analysing their translations demonstrates the variation in their choices, which constitute their own translation styles.

However, such research did not attract continuous research attention due to conflicting results. For example, in 2000, Baker discussed the existence of translator style: “it is as impossible to produce a stretch of language in a totally impersonal way as it is to handle an object without leaving one’s fingerprints on it” [25]; Baker suggested studying translator styles using forensic stylistics rather than literary stylistics [25]. According to Baker’s description, literary stylistics is generated by the choices that translators make consciously. On the other hand, forensic stylistics reflects unconscious linguistic habits, in which translators do not realise such linguistic preferences.

Baker suggested the existence of translator fingerprints, and she pioneered the research in this area and tried to identify some possible signatures for translators in their translations [25]. Although Baker’s study demonstrated the existence of translator stylometry, her study was limited in terms of computational linguistics analysis. Baker used translations of different languages and for different texts. The first translator translated from Portuguese and Spanish to English, while the second translator translated from Arabic into English. Furthermore, these translations are not for the same original texts. Such analysis left many open questions in terms of the translators’differences. Such differences would be assigned to translating from different original languages, or maybe variations in the original texts.

A subsequent study in 2011 considered attributing the translated text to their original author rather than the translator, thus considering the translator’s contribution to the text as noise [12]. The study investigated the use of semantic features to investigate authorship attribution of translated texts. The authors based their study on the expectation that the most significant effect of the translator is seen on the lexical and syntactic level, while the strongest influence of the author is on the semantic level. In other words, there was the expectation that translations and originals share the same semantic content.

In 2001, Mikhailov and Villikka questioned the existence of translators’ stylistic fingerprints [10]. That research was based on a parallel corpus of Russian fiction texts and their translations into Finnish. They used vocabulary richness, word frequencies, and favourite words. Their analysis shows that the language of different translations of the same text performed by different people is closer than that of the different translation by the same translator. Their finding concludes that despite the existence of some translators’ preference patterns, authorship existing techniques (which they evaluated) failed to identify translator styles. Using their words, “it appeared as if translators did not have a language of their own” [10]. Their conclusion was summed up in their title; “Is there such a thing as a translator’s style?”.

In 2002, Burrows proposed Delta analysis for authorship attribution. In his first trial, he worked on translations as well. In that study, Burrows examined fifteen translations of Juvenal’s tenth satire with English restoration poetry. With Delta distance, the output is a table containing authors ranked from the most possible author to the least possible author. Interestingly, Dryden’s rank on his translation was 9th out of 25. While Johnson style was correctly identified by Delta, Vaughan and Shadwell appeared significantly down the rank of their own translations.

Recently, in a number of studies by Rybicki and others in 2011 and 2012 [11, 26, 27], they investigated the problem of translator stylometry attribution by employing a well-known technique for authorship attribution called Burrows’s Delta [28], which is based on the z-score of the word frequencies. Burrows’s Delta has been used successfully for authorship attribution in multiple studies [29–33]. They submit the calculated z-score to Cluster Analysis to produce tree diagrams for a given set of parameters, such as the number of MFWs studied, pronoun deletion, and culling rate. Based on that culling rate, a decision is made to include a specific word in the analysis. Then, these results that produced a great variety of parameter combinations are used as input for a bootstrap procedure. Based on the generated tree, they analysed how these translations were grouped in the same branches.

In the first study, Rybicki employed this method for the investigation of the translator Jeremiah Curtin and his wife Alma Cardell’s contribution to his translations. Rybicki showed that *Memoirs of Jeremiah Curtin (1940)* is the work of his wife. In Rybicki’s investigation, those memoirs were clustered in a different branch with some other suspected literary works. The second study was by Heydel and Rybicki, who employed the same method to investigate if it can differentiate the collaborations between translators on a single literary work. They investigated a novel by Virginia Woolf *Night and Day*, which consists of 36 chapters. The first translator, Anna Kolyszko, died after translating the first 26 chapters, then another translator, Heydel, translated the remaining chapters. Their proposed method succeeded in clustering the translations according to their translators. Hydel and Rybicki highlighted that despite the success of these investigations, the detected translator signature may be lost if investigated in the context of different corpora.

In 2012, in another trial for translator stylometry attribution, Rybicki conducted a research study under the title of “The Great Mystery of the (Almost) Invisible Translator: Stylometry in Translation” [11]. The title reveals the challenge of identifying the translator of a piece of text. Rybicki’s approach failed to attribute texts to their translators using machine learning techniques and the use of most frequent words. He concluded that except for some few highly adaptative translations, the investigated method failed to identify the translator of the text, but it identified the author instead.

Rybicki found that in most of the cases, the translations were grouped based on the original author rather than the translators. For that study, he used a corpus of multiple language translations: Polish, English, French, and Italian translations. He tested each corpus translation group separately. Rybicki supports Venuti’s observation on translator’s invisibility, and concluded that multivariate analysis of most frequent word technique condemns translators to stylometric invisibility in the case of a large corpus of translations [11].

While some studies focused on human translations, other studies examined machine translation systems (MTS). However, the features used to identify translations generated by MTS were inherited from the way machine translation systems work. Features like “Gappy phrases”, where the phrase is composed of two sub-phrases that are separated by some text, are very easy for human generated text but constitute a difficult task for statistical machine translation. Another example is the Phrase Salad Phenomenon, where an observation of different phrases written fluently, with the inter-phrases displaying poor grammar or fluency connections [34]. Other stylistic features have also been deployed in this line of work, such as those by Ahroni et al [35] who used the number of part-of-speech n-grams and function words. The accuracy of classification using these features seems to correlate with the human evaluation score given to those translations. Translations with high human evaluation score were harder to be detected using these features. Our study however focuses on human-based translations, and therefore stylistic features used for human generated texts are evaluated.

### Complex networks and language network analysis

Text mining has been used in authorship attribution [36–38], text categorization [39], and sentiments analysis [40–42]. It aims at extracting patterns from natural language text rather than structured databases. The first step in text mining is to analyse the text in order to identify important features.

In this paper, we represent the text as a complex network, then we use complex network analysis to extract this network’s feature forming patterns for classification.

Complex network analysis has gained significant interest from researchers because of its ability to represent relationships among entities in a way that captures the interdependency in a sequence. Many research studies have benefited from using complex network analysis, such as, studies in occupational mobility, community detection, group decision making, social support, world political and economic system, and markets [43]. Network analysis has also shown favorable results in authorship attribution when examined by Mehri [44], [45] and Akimushkin [46].

Newman [47] defined a network as “a collection of points joined together in pairs by lines”; these points are referred to as vertices or nodes and the lines are referred to as edges. Examples of network mining problems include link prediction, link type prediction, discovery of communities of interest, and discovery of infrequent or unusual patterns. Networks can be analyzed through measurements on the global and local level. Local measures attempt to capture the global features of the network from local constructs. A widely used local measure is network motifs. Network motifs are used to uncover network structural design principles [48]. Network motifs have been successfully used by different researchers in biology [49, 50], game theory [51], electronic circuits [52], software [53], and language analysis [45, 54–57].

Among the complex networks studied in recent years, language networks gained the attention of many researchers. The choice of nodes and edges to represent linguistic features of interest were designed by researchers based on the distinguished features of each domain of application. Syntactic representations using language networks examples have been seen in [54, 55], and semantic representation examples are in [56, 57]. Co-occurrence networks -also known as adjacency networks- are a special type of syntactic representation where the nodes represent the words, and the edges represent the co-occurrence of two words within specific distance.

Co-occurrence networks have been used by a number of researchers for authorship attribution. Amancio [45] extracted global network features and used them for authorship attribution and genre identification problems as samples for stylometry problems, and the findings from this study recommended combining both classic statistical features with the network-based features to reach better accuracy. Akimushkin et al. [46] extended the analysis of word adjacency networks via dividing texts into equivalent length pieces before mapping them into networks. Then, global network features extracted from each of these networks were used to create a time series. Authorship identification tasks used features extracted from the time series obtained in the previous step.

Apart from global network features, local network features such as network motifs served as distinctive patterns in language networks. Biemann et al [56] used network motifs as signatures to distinguish between artificially generated language and natural language by analyzing the co-occurrence of graphs of verbs. Marinho et al [58] for example used network motifs identification in co-occurrence networks for authorship attribution. Amancio et al. [59] used global network features for authorship attribution with accuracy of 65% using feature selection. Without feature selection, using all of the global network features resulted in accuracy around 50% using the best classifier. The dataset used five novels for eight authors. Marinho et al [58] used the same dataset as in Amancio’s work, and employed network motifs, and the classification accuracy using the absolute frequencies of network motifs of size three was 57.5%. This accuracy has been achieved using lemmatization and without removing stop words before mapping the texts into networks.

Network-based methods are used for stylometry identification as they depend on the text structure rather than lexical features. Therefore, topic variations have low effect on the network structures [46]. Syntactical links captured by word-adjacency networks are language independent [45]. An interesting observation was the preprocessing stpdf required before mapping the text into a network for stylometry analysis. Lemmatization was an agreed step but stop words removal was not. Lemmatization is the process of reducing a word into its base form [60]. Amancio removed stop words before the lemmatization and network creation [45] as he suggests that they only serve a linking purpose and the edges already capture that. On the other hand, Marinho et al’s work recommended keeping the stopping words based on experimental results [58]. The accuracy dropped when they removed the stop words before forming the network.

## Corpora

In this study, we follow Baker’s definition of “Translator styles”: “a study of a translator’s style must focus on the manner of expression that is typical of a translator, rather than simply instances of open intervention. It must attempt to capture the translator’s characteristic use of language, his or her individual profile of linguistic habits, compared to other translators” [25]. Her definition of the style as a matter of patterning of linguistic behaviour is what we targeted in this research.

Moreover, we choose to work on translations from Arabic to English and Spanish to English. Arabic Language is the third most official language in the world after English and French; where it is the official language for 26 countries [61] with approximately 237 million native speakers in the world [62]. On the other hand, Spanish has been ranked as the second language per native speakers with 442 million speakers [63]. Spanish also is the official language for 20 countries [64].

Second language learners face difficulties when they learn a language that is derived from a different language family. For example, learning the German language for a native English speaker is not as difficult as learning Arabic. German and English languages belong to the same branch and subgroup in the taxonomy of language families. Both of them belong to the Western branch, the Germanic subgroup of the Indo-European family [65], while Arabic belongs to the Semitic subgroup of the Afro-Asiatic family [65]. Translating may pose similar difficulties between languages that belong to different language families; spaces of choices while mapping increase in this case. This degree of freedom while translating is expected to be less in the case of Spanish to English translations because both languages belong to the same language family (Indo-European). It is important to note here that Spanish and English originate from different main branches of the Indo-European Family as Spanish belongs to the Italic subgroup, while English belongs to the Germanic subgroup as mentioned earlier.

### 1st corpus: Arabic-to-English translations of the “*Holy Qur’an*”

The importance of the Arabic language can be understood with reference to its socio-political role as well as its religious role. Approximately 1.57 billion Muslims of all ages live in the world today [66] who read Qur’an on a daily basis as a part of their religious activities, which explains the reason for having millions of Muslims seeking to learn Arabic, the language of “The Holy Qur’an” which is the main religious text of Islam.

We choose to use the translation of the meanings of “The Holy Qur’an” as our corpus for this study. An important consideration for the choice of this text was the expectation that given its religious significance, there would be minimal difference in the translations; thus, it would be a tough translator stylometry challenge.

The translation of a religious book poses a significant challenge due to the requirement to convey the original message as accurately as possible. It adds more constraints and further limits the already limited translator freedom in translating the text. This type of challenge has an increased pressure when translating in two different cultural contexts. Mordai conducted an analysis of the strategies employed in three English translations of the holy Quran by Shakir, Pickthall, and Yousif Ali for handling cultural specific context [67]. Mordai found that the translators limited their strategies to four out of the seven available strategies defined by Ivir [68]: literal translation, definition, borrowing and addition. The translators didn’t use omission, substation or lexical creation at all. Furthermore, among the four strategies that they used, the literal translation choice was the most frequent one for each of these three translations. That supports our claim and choice for Holy Quran as a corpus for this study that incorporates an added level of challenge with the limited freedom translators have in expressing themselves through the target text.

Additionally, the availability of many translations for the same text provided a good source for evaluating the challenge of increasing the number of translators while trying to detect their translation stylometry. We obtained our corpus data from tanzil.net Tanzil is a quranic project launched in early 2007 to produce a highly verified unicode Quran text to be used in quranic websites and applications. This website offers different English translations for the meanings of Holy Qur’an. We use the seven translations of *Ahmed Raza Khan*, *Muhammad Asad*, *Abdul Majid Daryabadi*, *Abul Ala Maududi*, *Mohammed Marmaduke William Pickthall*, *Muhammad Sarwar*, and *Abdullah Yusuf Ali*.

The holy Quran is divided mainly into 114 surah (pl. suwar) which is also known by some as chapters, although they are not equal in length. The length of the surah varies from three ayat (verses) to 286 ayat. We will refer to them as chapters and verses in this study. Some Islamic scientists divided the Holy Quran into 30 parts (Juz’) which are roughly equal in length for easier citation and memorizing during the month. In this study, we are going to use the translations for the last six parts of the holy Qur’an which include 74 chapters. The size of each part for each translator is shown in Table 1.

**Table 1:** Number of words in the dataset for each translator of the Holy Qur’an.

Translator Name	Part25	Part26	Part27	Part28	Part29	Part30
khan	7427	4476	5974	5600	6182	5690
Asad	7326	7136	7261	7025	7499	6619
Daryabadi	6659	4105	5403	5100	5492	4942
Maududi	7310	4370	6255	5588	6291	5356
Pickthall	5340	4759	5384	5188	5477	4759
Sarwar	6831	4034	5654	5181	5784	5332
Yousif Ali	5950	5795	6019	5665	6265	5633

### 2nd corpus: Spanish-to-English translations of “*Don Quixote*”

“Don Quixote” is the short name of the famous Spanish “El ingenioso hidalgo don Quijote de la Mancha” which is translated to English as “The Ingenious Gentleman Don Quixote of La Mancha” by the Spanish writer Miguel de Cervantes. This Novel consists of two parts/books. The first part, published in 1605, consists of 52 chapters, while the second part, published in 1615, consists of 74 chapters. This novel has been translated to many languages including English. Among the different English translations available for this famous novel, we chose the following three translations because of the availability of digitized versions of these three translations: *Charles Jarvis* (1742), *John Ormsby* (1885), and *Thomas Shelton* (1612-1620). Statistics on the number of words in these translations are detailed in Table 2.

**Table 2:** Number of words per chapter in the dataset for each translator of Don Quixote.

Translator Name	Part one (52 chapters)	Part Two (74 chapters)
Jarvis	From 1700 to 8202	From 797 to 5530
Ormsby	From 1648 to 8304	From 823 to 5680
Shelton	From 1820 to 8921	From 759 to 5158

## Methodology: Using network motifs for detecting translator stylometry

We are targeting the translator style by detecting the repeated patterns in the translator writings. We employ complex network analysis for that purpose.

By representing the text as a word adjacency network, we can use network motifs to detect the existence of repeated patterns of ordered words -known in linguistics as ‘lexical chunks’- in the text.

In this research we evaluate both local and global network features. For evaluating local network features, we used network motifs of size three and four. The motifs are subgraphs that occur much more often than they do in random networks. The motifs usually consist of three, four or five nodes. For motifs of size three, subgraph with three connected nodes, we have only 13 distinguished possible subgraphs. For four connected nodes, we have 199 distinguished possible subgraphs, and 9364 possible subgraphs for five nodes. In this study, we use three -node and four -node motifs.

To evaluate global network features, we choose some of the common features like average of degree, density, clustering coefficient, transitivity, modularity, betweenness, characteristic path length, and diameter.

### Data pre-processing

For the data pre-processing step, the Natural Language Toolkit NLTK of Python programming language had been used. First the text is cleaned from anything except alphanumeric and decimal digits. Then, each sentence is tokenized into words. After that, these words are lemmatized to their lemmas. Then, each lemma of these words is lowercased. By completing this data pre-processing stage, all of the occurrences of the same word (including their inflections) can be identified and grouped together during the formation of the word adjacency network. Following the findings of Marinho et al [58], we didn’t remove the stop words before mapping the texts into networks.

### Network formation

To establish the word-adjacency network from the dataset, co-occurrence between two words is counted if these two words occurred within the same sentence. For the first corpus, each Ayah is considered as a single sentence. Each word is represented by a node, and each ordered word adjacency is represented by an edge (a link that connects two nodes) going from the first occurring word to the following word. The frequency of two adjacent words is counted and represented by edge labels. The edges here represent an “occurring- before” binary relationship.

### Counting network motifs size three and size four

To illustrate how we can extract these 13 motifs, we give an example of a network generated using a sample translation by “Yousif Ali” for chapter 112 from the Holy Qur’an. The sample text is “*Say: He is Allah, the One and Only; (1) Allah, the Eternal, Absolute; (2) He begetteth not, nor is He begotten; (3) And there is none like unto Him. (4)*”.

**Fig 1:**
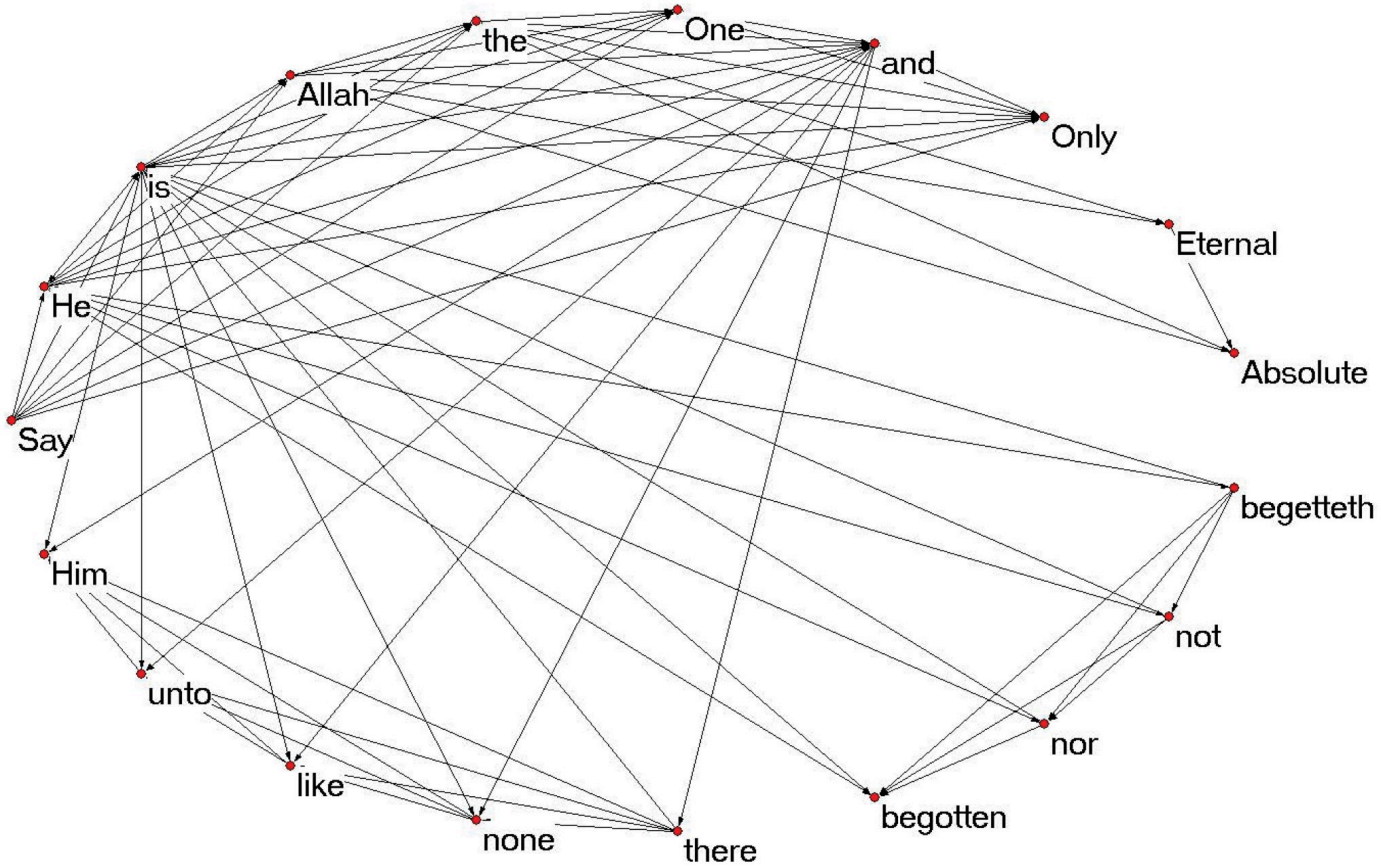
Network example: “Yousif Ali” for chapter 112, nodes represent the words, directed edge represents “occurring-before” relationship.

**Fig 2:**
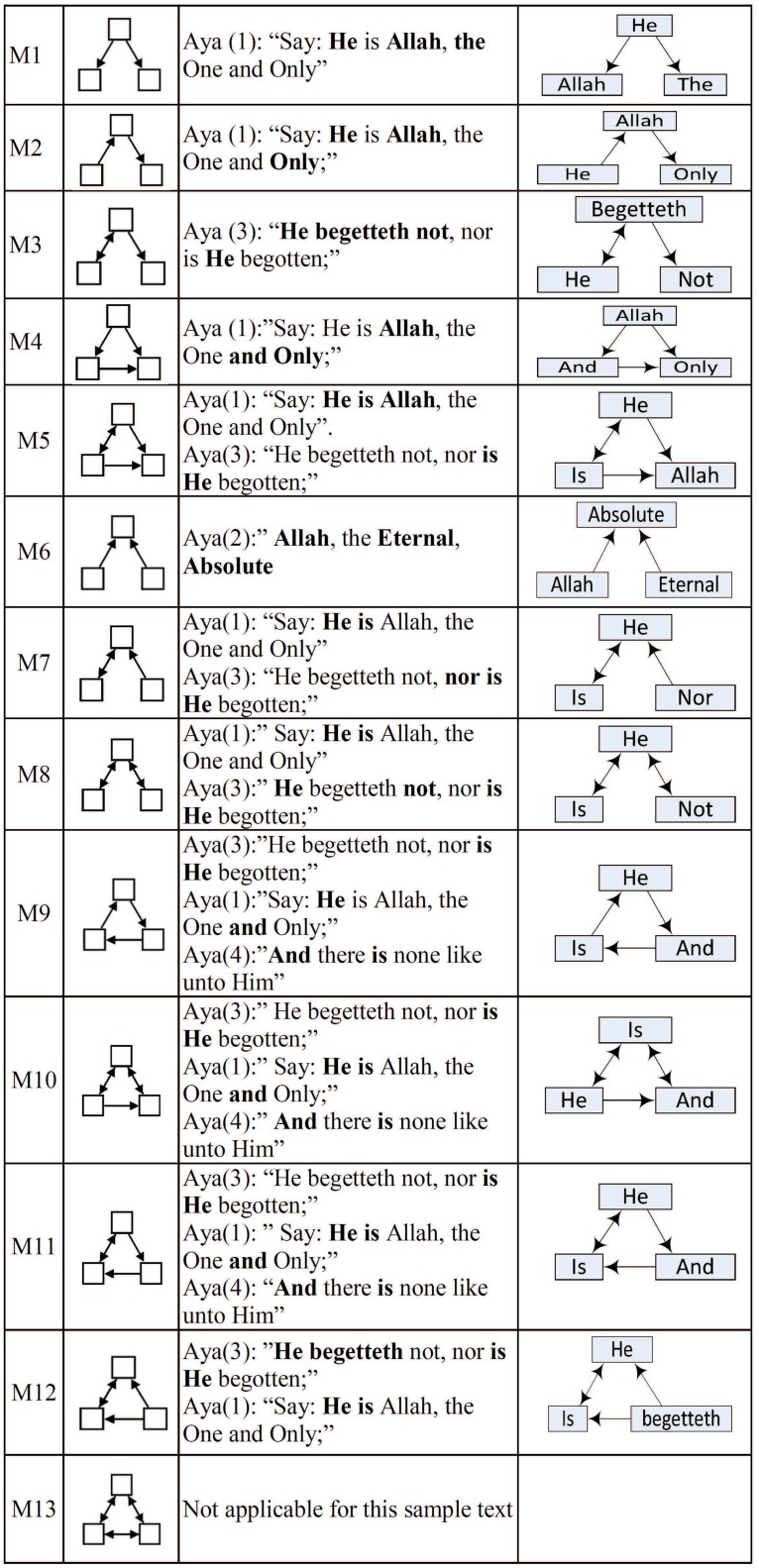
All possible 3-nodes connected subgraph.

The network formed to represent this sample text is shown in Fig 1, and examples of the extracted motifs are shown in Fig 2.

For example, motif 7 (M7) represents the relationship between three nodes; a two way relationship between the left and upper nodes, and one way relationship between the right and upper nodes. The first relationship is represented by the ordered appearance of words “is” and “He” in Aya (3), and the other direction where “He” before “is” in Aya (1). The second relationship is represented by the ordered appearance of words “nor” and “He” in Aya (3).

In motif 8 (M8), the first relationship that appeared in motif 7 is the same, while we have another two way relationship between the upper and right nodes, represented in Aya (3) where the word “He” appeared once before “not” and the second time after it.

For the purpose of counting all occurrences of all possible motifs of size three and size four, we used a motif detection tool that is called Mfinder [69] which uses an algorithm that is explained in detail in Kashtan et al’s research in 2004 [70].

### Global network features

Among the different global network features, we chose nine of them to be the classification attributes. All of these measures were evaluated using *brain connectivity toolbox* [71]. Their definition according to this toolbox is as follows:

- Average degree: It is the average of weighted degree which is calculated using the following equation
  $$\begin{array}{c} k_{i}^{w}=\sum_{j\in N}w_{ij} \end{array}$$ (1)
  Node degree is the number of links connected to the node. In directed networks, the indegree is the number of inward links and the outdegree is the number of outward links.
- Assortativity: The assortativity coefficient is a correlation coefficient between the degrees of all nodes on two opposite ends of a link. A positive assortativity coefficient indicates that nodes tend to link to other nodes with the same or similar degree.
- Density: The fraction of present connections to possible connections. Connection weights are ignored in calculations.
- Clustering coefficient: The clustering coefficient is the fraction of triangles around a node (equiv. the fraction of node’s neighbors that are neighbors of each other
- Transitivity: The transitivity is the ratio of ‘triangles to triplets’ in the network (an alternative version of the clustering coefficient).
- Modularity: The modularity is a statistic measure that is used to quantify how the network can be divided into optimal community structure, which is subdivision of the network into nonoverlapping groups of nodes in a way that maximizes the number of within-group edges, and minimizes the number of between-group edges.
- Betweenness: Node betweenness centrality is the fraction of all shortest paths in the network that contain a given node. Nodes with high values of betweenness centrality participate in a large number of shortest paths.
- Characteristic path length: The characteristic path length is the average shortest path length in the network.
- Diameter: The diameter is the maximum eccentricity.

## Experiments

### Experiment I: Is there such a thing as a translator’s style

The first question that our research aimed to address was the existence of translator individual styles. In 2001 Mikhailov and Villikka [10] argued that translators do not have a linguistic style of their own. In their research, they used three typical authorship attribution features, namely, vocabulary richness, most frequent words and favourite words to prove their claim. In response to this paper, and as a first step, we reapplied the same method and features on the Arabic dataset.

1. Vocabulary Richness
  Vocabulary richness can be evaluated using different methods. Mikhailov and Villikka [10] used three different measures of vocabulary richness based on a multivariate approach that were originally introduced by Holmes in 1991 [72] then modified by Holmes and Forsyth in 1995 [73]. These three measures are:
  - R-index is a measure suggested by Honore (1979). This measures targets (hapax legomena) which means words that are used only once in the text. The higher the number of words used only once in the text, the higher the R value.
    $$\begin{array}{c} R=\frac{100LogN}{1-\frac{V1}{V}} \end{array}$$ (2)
    where N is the text length of N words, V1 is the number of words used exactly once in the text, and V is the number of different words;
  - K-index is a measure that was proposed by Yule (1944). The measure monotonically increases as the high-frequency words in the text increases.
    $$\begin{array}{c} K=\frac{10^{4}\left(\sum_{i=1}^{\infty }i^{2}V_{i}-N\right)}{N^{2}} \end{array}$$ (3)
    where V_*i*_(*i* = 1,2,…) is the number of words used exactly *i* times in the text,
  - W-index is originally proposed by Brunet (1978), who suggested that this measure is not affected by text length, and it is author specific. W-index increases when the number of different words increases.
    $$\begin{array}{c} W=N^{V-a} \end{array}$$ (4)
    where *a* is a constant ranges from 0.165 to 0.172. The methodology for selecting the value of (a) was not explained in the research by Mikhailov and Villikka [10]. For this reason, we employed the value of 0.172 which was used in their research.
2. Most Frequent Words
  F-Index is used to measure the closeness of most frequent words as it reflects a correlation between two pieces of texts. The two targeted texts are compared by selecting the 40 most frequent words from their word lists. Then, the F-Index is calculated by adding three points for each word with close relative frequency, two points for each word with different relative frequency, and one point for each word with quite different relative frequency. One point is deduced for each word which is absent in the other list. We applied this method on lemmatized word lists for each text in our dataset.
  To calculate the F-Index, we needed to define threshold for close relative frequency, different relative frequency, and quite different relative frequency, which were not defined in Mikhailov and Villikka research [10]. To do that, we divided the distance between the minimum frequency and maximum frequency to three equal parts, the first section represents low difference area, the middle two sections represent medium difference, and the last section represent high difference area. If the difference between the frequency of the same words in the two texts occur in the low difference area, that means they are relatively close to each other, and F-index is incremented by three. If the difference occurs in the medium difference area, it is considered as being quite different, and the F-Index is incremented by two. Otherwise, the F-Index is incremented by one.
3. Favorite Words
  To calculate the Favorite words, the relative frequency for each word is calculated for the whole corpus. The output of this first step is a sequence of word-frequency pairs. This step is repeated for each text. At the conclusion of this process, the sequences are merged and sorted on frequency. The sequences with the highest frequencies are stored in a list. The threshold used for this filtering process is called Alpha. We tested different values for Alpha to choose a suitable one.
  We then re-applied the same method for F-Index, which was described in the most frequent words subsection to compare the two filtered lists for the two texts that we wanted to compare. Although the list size in the most frequent words method is predefined with the top 40 most frequent words, for FW-Index the size changes based on changing alpha. To define a threshold for the condition “where word freq in a text is much higher than in the corpus” [10], we have *Fc*(*w*_1_) representing the frequency of *word*_1_ in the corpus, *F*_*i*_(*w*_1_) represents frequency of word *w*_1_ in *text*_*i*_. If (*Fi*(*w*1_)_/*Fc*(*w*_1_)) is greater than alpha, then its frequency is much higher than in the corpus.
  To define an appropriate value for alpha, we used two parts translated by two different translators; where we have part 25 translated by Ahmed Raza and Pickthall, and part 27 is translated by sarwer and yousifali. Table 3 represents the affection of alpha choice on FW-Index, it also shows that choosing alpha as twice or three times the frequencies introduces an acceptable number of words in the list and FW-Index. So, we chose alpha as 3, as it complies with more than 2 for the condition “where word freq in a text is much higher than in the corpus” that had been described in Mikhailov and Villikka [10].

**Table 3:** Affection of choosing alpha on the number of words in the coincidences lists of FW-Index for two tested texts.

Alpha	Part 27		FW-Index
	Sarwer (5654 words)	Youasif ali (6019 words)	
4	347	467	75
3	448	538	103
2	573	694	167
1.5	691	802	227

In this experiment, we aimed to evaluate Mikhailov and Villikka approach using our dataset [10]. We found that “chapters” will be too small to be used with these measures, as some of them have the limitation of working with text size of 1000 words or more. So, we worked with the level of parts of the Holy Qur’an. More details about the possible divisions of the Holy Qur’an were explained earlier in the data section. Therefore, we used seven translations for six parts of the Holy Qur’an in this experiment.

For vocabulary richness: R-Index, K-Index, and W-Index were calculated for each text. Then the results for these calculations were used to compare and analyze the similarities and differences between translations by the same translator with translations for the same text. Unlike the most frequent word index and favorite word index, which are calculated between two different pieces of text, vocabulary richness indicators can be calculated for each piece of text separately. The objective of this experiment was to identify if vocabulary richness for the same translator is maintained over different translations, or it changes according to the original texts.

For the most frequent and most favourite words, we cannot evaluate a single text each time; as the proposed measures are used to measure similarities between two texts. Therefore, for the most frequent words, we calculated this measure for all possible combinations for the existing dataset. First, we calculated all the pairs of translations by the same translator. For example, for translator Asad, we calculated the most frequent words measure, F-Index, for (part25-part26), then for (part25-part27), then for (part 25-part 28),…etc. After measuring these for all translators, we evaluated the F-Index for different translators for the same original text. So, For Part25, we calculated F-Index for (Asad-Daraybadi), (Asad,Maududi), (Asad-Pickthall),…etc. Then, all of these results were used to identify whether the most frequent words measure is affected by the translator style or the original text. The same procedure is used for evaluating the favorite words measure.

#### Results and discussion of Experiment I

Detailed results for R-Index, K-Index and W-Index are shown in Table 4. For Vocabulary Richness, the three used measures are highly affected by the original text.

**Table 4:** Vocabulary richness measures.

Translator Name	R-Index						K-Index						W-Index					
	P25	P26	P27	P28	P29	P30	P25	P26	P27	P28	P29	P30	P25	P26	P27	P28	P29	P30
khan	822.19	864.64	901.84	744.06	876.56	890.48	130.82	123.86	129.06	134.52	115.02	141.02	14.20	13.41	13.39	14.33	13.36	13.14
Asad	812.08	863.62	903.85	802.34	921.75	895.26	81.70	79.09	83.67	89.62	75.67	84.77	12.89	12.61	12.41	13.07	12.10	12.14
Daryabadi	811.20	849.03	918.87	780.07	952.72	937.62	125.21	124.14	137.23	131.87	111.91	133.41	13.92	13.18	13.03	13.80	12.70	12.67
Maududi	791.12	852.45	902.81	806.51	934.09	951.88	106.52	103.64	117.89	117.14	109.25	123.63	13.80	13.19	13.13	13.59	12.71	12.59
Pickthall	813.36	937.52	905.89	768.45	928.14	937.52	121.72	126.09	138.34	123.55	102.87	126.09	13.71	12.48	13.08	13.94	12.65	12.48
Sarwar	773.02	827.20	848.06	757.14	899.48	907.85	118.48	119.31	123.51	128.40	106.65	130.98	13.91	12.93	13.23	13.83	12.70	12.71
Yousif Ali	823.53	845.97	901.53	793.74	896.07	906.08	105.01	102.00	118.59	118.37	98.57	118.29	13.52	12.99	12.76	13.58	12.53	12.42

P in the column titles refers to Part. P25 refers Part 25, P26 refers to Part26, etc…

The R-Index didn’t reflect an individual translator style; translations are mostly affected by the original text. Both K-Index and W-Index also didn’t reflect individual translator styles. However, Asad had lower K-index for all translations, and Khan had the highest W-index values for all translations. This implies that both K-Index and W-Index can show individual styles for some special cases, which required further analysis.

For Most Frequent Words, Table 5 shows F-Index for translations of the same text. These numbers (F-Index) reflect how close the most 40 frequent words are in each of these translations, while Table 6 shows F-Index for two translations for the same translator.

**Table 5:** Most frequent words index—For the same part.

Part Number	AS-DR	AS-MD	AS-PK	AS-KH	AS-SR	AS-YA	DR-MD	DR-PK	DR-KH	DR-SR	DR-YA	MD-PK	MD-KH	MD-SR	MD-YA	PK-KH	PK-SR	PK-YA	KH-SR	KH-YA	SR-YA
Part25	69	90	70	69	74	85	73	95	70	59	79	78	94	85	95	70	64	89	69	80	80
Part26	64	85	75	84	79	80	67	100	70	72	80	72	94	80	99	75	73	85	84	90	84
Part27	59	80	69	59	74	80	79	95	70	65	80	79	85	75	89	85	75	100	70	85	85
Part28	63	80	70	69	74	80	73	95	70	67	79	78	95	85	89	85	84	100	84	89	84
Part29	69	79	74	74	74	70	89	105	75	74	89	89	95	85	100	80	75	95	80	85	70
Part30	75	80	80	69	84	80	75	95	70	70	95	85	100	85	85	80	75	95	90	75	75

Abbreviations of translators’ names are used in this table: AS for Asad, DR for Daryabadi, MD for Maududi, PK for Pickthall, KH for Khan, SR for Sarwar, YA for Yousif Ali.

**Table 6:** Most frequent words index—For the same translator.

Translator Name	P25-P26	P25-P27	P25-P28	P25-P29	P25-P30	P26-P27	P26-P28	P26-P29	P26-P30	P27-P28	P27-P29	P27-P30	P28-P29	P28-P30	P29-P30
Asad	100	85	89	105	85	90	100	100	80	84	85	75	89	73	85
Pickthall	90	95	79	100	75	90	100	95	70	79	99	75	84	64	95
Yousif Ali	85	85	74	80	80	89	100	75	70	84	85	80	69	64	90
Khan	100	85	74	105	85	85	90	100	85	64	90	75	74	74	90
Daryabadi	100	100	94	100	85	95	105	100	85	89	90	75	94	80	100
Maududi	95	85	79	95	85	80	100	90	85	79	85	75	84	83	100
Sarwar	90	80	84	85	90	80	95	90	90	79	95	85	89	99	105

P in the column titles refers to Part. P25 refers Part 25, P26 refers to Part26, etc…

The average of F-Index for translations for the same text is 80.19 with a STD of 10.01 while the average for F-Index for translations for the same translator is 86.94 with a STD of 9.85. The average F-index for translators over different translations is higher than those between different translations of the same original text. However, the high STD, which is larger than the difference between the two averages, prevent us from generalizing the finding of assuming that translator choices have slightly higher effect compared to the original text effect on the favorite words index. Therefore, a closer look at F-index for each translator is the next important step.

With regard to the measure of Favourite Words, Table 7 shows FW-Index for translations of the same text, where FW-Index reflects how close the favourite words lists are in a binary comparison of translations. Table 8 shows FW-Index for translations for the same translator. The results showed that the average of FW-Index for translations of the same text is 110.93 with a STD of 31.28 while the average for FW-Index for translations of the same translator is 71.61 with a STD of 16.70. These tables show that favourite words list doesn’t reflect a translator signature. The translation is influenced more by the original text than translator individual styles.

**Table 7:** Favorite words index—For the same translator.

Translator Name	P25-P26	P25-P27	P25-P28	P25-P29	P25-P30	P26-P27	P26-P28	P26-P29	P26-P30	P27-P28	P27-P29	P27-P30	P28-P29	P28-P30	P29-P30
Asad	97	80	86	94	79	75	88	64	67	71	91	98	65	66	67
Pickthall	87	76	56	70	58	66	80	71	57	55	82	73	58	60	81
Yousif Ali	54	50	52	61	41	51	64	61	61	54	94	78	46	53	96
Khan	93	74	67	83	61	77	82	92	87	76	94	82	63	62	106
Daryabadi	94	89	71	86	73	84	103	78	83	77	98	91	68	65	100
Maududi	50	55	43	58	37	50	49	55	44	35	73	53	42	43	69
Sarwar	99	81	75	84	69	78	76	80	76	63	95	76	67	55	96

P in the column titles refers to Part. P25 refers Part 25, P26 refers to Part26, etc…

**Table 8:** Favorite words index—For the same part.

Part Number	AS-DR	AS-MD	AS-PK	AS-KH	AS-SR	AS-YA	DR-MD	DR-PK	DR-KH	DR-SR	DR-YA	MD-PK	MD-KH	MD-SR	MD-YA	PK-KH	PK-SR	PK-YA	KH-SR	KH-YA	SR-YA
Part25	55	98	67	64	57	88	84	140	61	56	79	66	83	82	109	70	65	70	87	76	67
Part26	69	120	88	88	82	83	117	183	74	77	117	112	109	115	115	88	89	108	104	88	91
Part27	88	139	101	101	81	95	127	182	85	90	109	118	112	111	122	107	85	110	92	97	103
Part28	82	135	98	98	92	113	128	147	87	79	117	108	114	122	132	104	94	106	115	102	115
Part29	97	146	97	97	93	125	165	199	80	110	165	125	119	141	152	110	130	135	123	100	123
Part30	124	158	142	142	109	137	171	224	117	131	157	166	134	153	176	136	145	152	127	120	140

Abbreviations of translators’ names are used in this table: AS for Asad, DR for Daryabadi, MD for Maududi, PK for Pickthall, KH for Khan, SR for Sarwar, YA for Yousif Ali.

**Fig 3:**
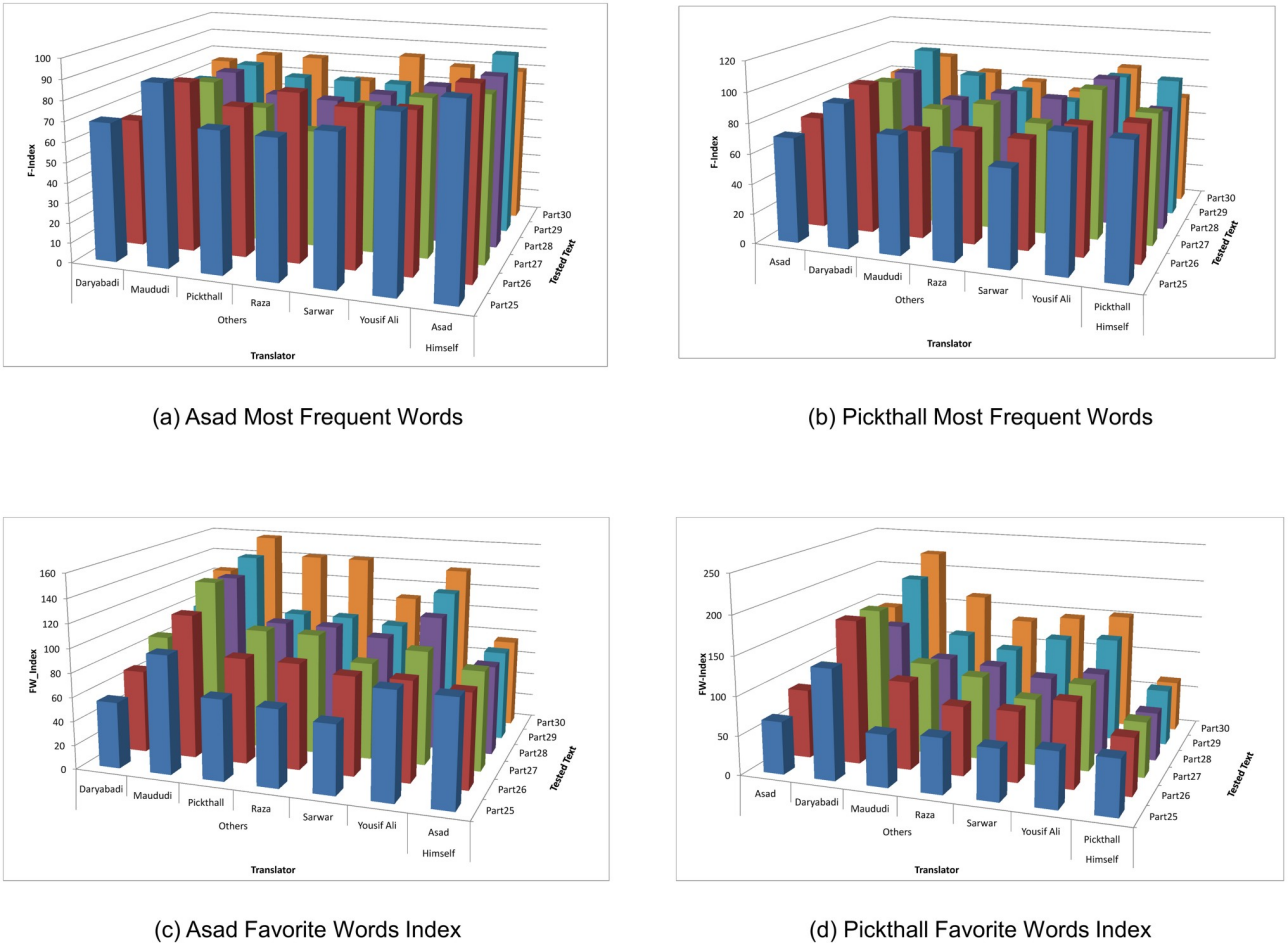
Comparison between most frequent words index and favorite words index for translators Asad and Pickthall.

To obtain meaningful information from Tables 5 and 6, we extracted values for one translator, Asad, to compare the closeness between his own writing with translations that are written by others for the same original text. This comparison is shown in Fig 3(a). The first six columns represent the F-Index for the most frequent words for the six translators, while the last column represents the average of F-Index for Asad writings. For example, for Text “Part 25”, we calculate the average of F-Index for “Part 25” and “Part 26”, “Part 25 and Part 27”, “Part 25 and Part 28”, “Part 25 and Part 29”, and “Part 25 and Part 30”. The same method is repeated for all other texts. Although Fig 3(a) shows that the F-index for Asad-to-himself is higher than Asad-to-others, by repeating the same analysis on another translator Pickthall, we found the F-Index for Pickthall-to-himself is average compared to Pickthall-to-others F-index as shown in Fig 3(b).

We used the same method of analysis employed for measuring the most frequent words to extract meaningful information from Tables 7 and 8; We analyzed the results for translators Asad and Pickthall. Fig 3(c) and 3(d) show that FW-Index translators-to-their selves is considered slightly lower than FW-Index of translators- to-others. In conclusion, the favorite words list cannot be used to identify translators’individual styles; the translation is affected by the original text rather than the translator’s choices.

### Experiment II: Vocabulary richness measures as translator stylometry features

To evaluate the effectiveness of the vocabulary richness criterion in measuring translator stylometry, we used the idea of classifying texts (as instances) into their translators (as classes) based on vocabulary richness (as attributes). Working on the level of parts for the Holy Qur’an as the instances gave us only 6 instances (parts)/ per class (translator). For that reason, we chose to work on the level of chapters, so that resulted in 74 instances /class.

For this experiment, we used five vocabulary richness measures as attributes: which are N, V, R-Index, K-index, and W-Index. Their description is discussed in Experiment I section 1.

We use one of the most studied classification algorithms in the literature: these are the decision tree C4.5 and support vector machine (SVM). We used their implementation in WEKA “data mining software”. For C4.5, which is a decision tree based classification algorithm developed by Quinlan in 1993 [74], we used pruned weka.classifiers.trees.J48. For SVM, we used weka.classifiers.functions.SMO; which is based on Sequential Minimal Optimization algorithm for support vector machine [75] [76].

As we have seven translators, we analysed them in pairs using binary classifiers. We used ten-fold cross-validation to evaluate the classifiers.

The results of this experiment will be presented in conjunction with the results of experiment III to allow comparison.

### Experiment III: Using network based features for detecting translator stylometry

In this experiment, we had three groups of features: the first one is 13 attributes which are all possible network motifs of size three, the second group is 199 attributes which are all the possible network motifs of size four, and the third group is nine attributes, which are the selected global network features that we used. All of these three groups of features were used as attributes for two types of classifiers, C4.5 and SVM. We fed these classifiers with 74 instances, chapters, for each of the seven translators. We used 10 folds cross-validation for evaluation.

#### Results and discussion of Experiments II and III

The results of Experiment II and III with the first dataset “Holy Qur’an”, presented in Table 9, showed that both vocabulary richness measures and network motifs did not provide satisfactory results neither using C4.5 nor SVM. The overall average accuracy of vocabulary richness was 55.12% using C4.5 and 54.31% using SVM, and the average accuracy of network motifs of size three was 49.84% using C4.5 and 49.81% using SVM.

**Table 9:** Classification results for vocabulary richness measures, network global features, motifs size three and motifs size four as translator stylometry features for the 1st corpus.

The Translators’Names	Vocabulary Richness		Global Features		Motifs Size Three		Motifs Size Four	
	C4.5	SVM	C4.5	SVM	C4.5	SVM	C4.5	SVM
Asad-Daryabadi	76.35%	77.70%	50.68%	60.14%	57.43%	54.05%	58.11%	52.70%
Asad-Maududi	71.62%	74.32%	47.97%	50.00%	55.41%	52.70%	53.38%	50.68%
Asad-Pickthall	81.76%	70.95%	52.03%	60.81%	52.03%	52.70%	51.35%	52.70%
Asad-Raza	76.35%	82.43%	54.05%	55.41%	54.73%	54.05%	52.03%	52.70%
Asad-Sarwar	72.30%	67.57%	50.68%	54.73%	58.11%	52.70%	54.73%	51.35%
Asad-Yousif Ali	68.92%	66.89%	45.27%	53.38%	54.73%	52.70%	54.05%	51.35%
Daryabadi-Maududi	50.00%	50.00%	47.30%	46.62%	47.30%	49.32%	46.62%	47.97%
Daryabadi-Pickthall	47.30%	39.19%	47.30%	43.92%	47.30%	41.89%	47.30%	44.59%
Daryabadi-Raza	47.30%	49.32%	61.49%	51.35%	45.95%	50.68%	46.26%	53.06%
Daryabadi-Sarwar	46.62%	50.68%	47.30%	43.24%	47.30%	42.57%	47.30%	54.05%
Daryabadi-Yousif Ali	51.35%	53.38%	47.30%	50.68%	47.97%	50.00%	50.00%	43.92%
Maududi-Pickthall	43.92%	46.62%	45.95%	48.65%	47.30%	50.68%	45.95%	49.32%
Maududi-Raza	45.27%	45.27%	54.73%	50.68%	47.30%	50.68%	47.30%	54.73%
Maududi-Sarwar	47.30%	45.95%	47.30%	43.92%	47.30%	50.00%	47.30%	42.57%
Maududi-Yousif Ali	47.30%	40.54%	46.62%	35.14%	47.30%	46.62%	47.30%	43.24%
Pickthall-Raza	46.62%	45.95%	67.57%	53.38%	47.97%	50.00%	49.32%	45.27%
Pickthall-Sarwar	46.62%	48.65%	47.30%	43.24%	47.30%	44.59%	47.30%	48.65%
Pickthall-Yousif Ali	45.95%	47.30%	47.30%	48.65%	50.68%	52.70%	47.97%	52.03%
Raza-Sarwar	49.32%	47%	55.41%	52.70%	46.62%	45.27%	47.30%	51.35%
Raza-Yousif Ali	47.97%	47.97%	58.78%	47.97%	47.30%	51.35%	46.62%	51.35%
Sarwar-Yousif Ali	47.30%	43.24%	47.30%	43.92%	49.32%	50.68%	49.32%	47.97%
Average	55.12%	54.31%	50.93%	49.45%	49.84%	49.81%	49.37%	49.60%
STD	12.58%	12.74%	5.85%	6.08%	3.88%	3.58%	3.34%	3.73%
*P*(*T* ≤ *t*) one-tail *H*0: *μ*(*VR*) ≤ *μ*(*M*3) *H*1: *μ*(*VR*)>*μ*(*M*3)	0.0105	0.0392						

On the other hand, different results were achieved with the second dataset “Don Quixote”. Although vocabulary richness failed again with average accuracy of 53.59% and 57.49%, the network motifs achieved acceptable results of 77.14% using SVM and 69.46%. These results are detailed in Table 10.

**Table 10:** Classification results for motifs of size three and vocabulary richness as translator stylometry features for the 2nd corpus.

Features		Motifs Size Three with Nodes and Edges		Vocabulary Richness		Motifs and Vocabulary Richness	
Data Set	Translators	C4.5	SVM	C4.5	SVM	C4.5	SVM
First Part (52 Chapters)	Jarvis- Shelton	83.65%	87.50%	58.65%	56.73%	76.92%	89.42%
	Jarvis- Ormsby	49.04%	67.31%	50.00%	46.15%	48%	68.27%
	Ormsby- Shelton	69.23%	76.92%	54.81%	59.62%	64.42%	81.73%
Second Part (74 Chapters)	Jarvis- Shelton	85.14%	88.51%	61.49%	67.57%	85.81%	91.22%
	Jarvis- Ormsby	55.41%	58.78%	43.92%	51.35%	53.38%	60.81%
	Ormsby- Shelton	74.32%	83.78%	52.70%	63.51%	82.43%	85.81%
Average		69.46%	77.14%	53.59%	57.49%	68.51%	79.54%
STD		14.73%	11.95%	6.27%	7.86%	15.67%	12.30%
*P*(*T* ≤ *t*) one-tail *H*0: *μ*(*M*3) ≤ *μ*(*VR*) *H*1: *μ*(*M*3)>*μ*(*VR*)				0.0054	0.0007		

For the first corpus, we evaluated the three network-based group of features compared to vocabulary richness, which represents the classic stylometry features. We used the findings from this comparison in Table 10 to make a decision on which network-based features to include in our further analysis. We excluded global network features due to their high variation compared to the network motifs. Then, since both network motifs of size three and size four achieved average accuracy around 49%, network motifs of size three group was chosen as it only uses 13 motifs (features) to represent. Therefore, we limited our analysis for the second corpus to vocabulary richness and network motifs.

One tail Paired Significance T-Test between vocabulary richness and network motifs of size three showed that the null hypothesis of *H*0: *μ*(*M*3) ≤ *μ*(*VR*) should be rejected for both C4.5 and SVM, and the alternative hypothesis of *H*1: *μ*(*M*3)>*μ*(*VR*) should be accepted.

Results of Experiment III with the first dataset “Holy Qur’an” did not match our expectation. This expectation was based on our previous paper, where we attempted to classify two translators using network motifs [77]. However, here we attempted to use 7 translators. The results were beyond our expectation. Both network motifs and network global features failed to identify translator stylometry as shown in Table 9.

We found that as the size of the network varied in a wide range among instances and between each other, the number of the subgraphs varied widely as well. In the first dataset, the network sizes varied from 19 nodes (as in Pickthall:Chapter109, Sarwar:Chapter112, Yousif Ali:Chapter112) to 597 (as in Asad: Chapter42), and the number of edges varied from 64 (as in Sarwar: Chapter112) to 29851 (as in Asad:Chapter42). As the number of subgraphs is highly affected by network size, using the values of motifs count directly misled the classifiers. The classifiers failed to detect the order/rank information. In the second dataset, where variations in the sizes of the chapters were not as wide as in the case of the first dataset, network motifs were able to perform better and to achieve good classification accuracy.

### Experiment IV: Non-parametric scaling for detecting translator stylometry

The accuracy levels obtained by the previous experiment were much lower than expected. We carefully investigated the problem and identified that the main problem lies in the variations of magnitude among the different instances. For example, a translator *A*1 may have 20 and 50 motifs of type 1, while translator *A*2 may have 40 and 70 of the same motif type. A classifier that attempts to establish a relationship based on a threshold as in the case of *A*1 ≤ 20 *and* *A*2 ≤ 40 is incapable of noticing that *A*1 has always the lowest number of motifs. Replacing the raw counts with the rank (some sort of non-parametric scaling), provides information on the position occupied by each translator for a given instance.

In the literature of authorship attribution, a method to enhance the performance of both network based features and vocabulary richness features has been proposed by Akimushkin et al. [78]. They proposed a method to select which words to be represented by nodes before mapping the text into a network. Employing similarity index to identify the most relevant words to be considered when calculating the network metrics boosted the classification accuracy. Multi-dimensional scaling (MDS) was also used for dimensionality reduction of the dissimilarity metrics before using classification.

On the other hand, as we deal with translations, where we have parallel translations linked to the same source text, we propose a method to capture the translators’signature by maintaining the link represented by the original text and use non-parametric scaling within each group of parallel translations.

### Method III

To express the discussed relationship, for the first corpus, we grouped the translated text based on their original sources; the seven parallel translations of the first chapter(surah) in our analysis, chapter 41, are grouped in the first group, the seven translations of chapter 42 are grouped in the second group, and so on. Then, within each group, we compared motif id “1” for all translators, and replaced the frequency with rank of the translator. For example, if for a piece of text M3 is 10 for Author A1, 20 for Author A2, 30 for Author A3, we replace these frequencies with “3” for Author A1, “2” for Author A2 and “1” for Author A3. Here “1” for Author A3 means that Author A3 ranks on M3 for this piece of text is the highest. In the case of tie, the two equal values receive equal rank, and one rank step is skipped in the order.

Similarly, the same ranking approach is applied to the vocabulary richness measures that we described in Method II to investigate the performance of network motifs and vocabulary richness.

We applied the proposed method for two categories of features: the first was network motifs, and the second was the vocabulary richness.

For the network motifs, both motifs of size three and size four were included. We also used the same classification algorithms and dataset as in the previous experiment. We evaluated the attributes in five groups. The first group contains 13 attributes (all the possible 13 motifs of size three). The second group contains 15 attributes, which are the same as the first group in addition to the number of nodes and edges for each instance. The third group contains 199 attributes (all the possible 199 motifs of size four). The fourth group contains 201 attributes which are the 199 attributes of the third group in addition to the number of nodes and edges. The fifth group contains 214 attributes which are all the possible motifs of size three and size four in addition to the number of nodes and edges.

For the vocabulary richness category, five features were included: R-Index, K-Index, W-Index, N, and V, which are described in the methodology section.

#### Results and discussion of Experiment IV

The average of the classifiers that were built using the five groups of network motifs attributes introduced acceptable results as shown in Table 11. They ranged from 75% to 79.02%. Moreover, some of the individual classifiers performed very well with up to 97.97% accuracy as in the case of translator (Asad-Pickthall). On the other hand, some pairs of translators couldn’t be distinguished from one another. The five groups of attributes failed to differentiate between them. This happened with three pairs of translators (Daryabadi-Pickthall), (Maududi-Yousif Ali), and (Maududi-Sarwar). Generally, SVM classification algorithm outperformed C4.5 decision tree. Comparing the five groups of attributes to each other, we found that the best accuracy was achieved by the fifth group (all the motifs of size three and four and the number of nodes and edges) using SVM classifier. However, this accuracy was not much higher than found in all the other groups in the case of SVM.

**Table 11:** Classification results for applying ranking to motifs of size three and size four, and vocabulary richness as translator stylometry features for the 1st corpus.

Features Type	Network Motifs										Vocabulary Richness	
Translators’Names	Motifs Size Three		Motifs Size Three with Nodes and Edges		Motifs Size Four		Motifs Size Four with Nodes and Edges		Motifs Size Three and Size Four with Nodes and Edges			
	C4.5	SVM	C4.5	SVM	C4.5	SVM	C4.5	SVM	C4.5	SVM	C4.5	SVM
Asad-Daryabadi	96.62%	97.30%	96.62%	97.30%	96.62%	96.62%	96.62%	96.62%	93.92%	97.30%	97.30%	96.62%
Asad-Maududi	89.86%	85.81%	88.51%	91.22%	85.81%	87.16%	85.81%	87.16%	88.51%	87.16%	91.22%	93.92%
Asad-Pickthall	97.97%	97.30%	97.97%	97.30%	91.22%	95.95%	91.22%	95.95%	97.30%	95.95%	95.95%	95.95%
Asad-Raza	79.73%	86.49%	81.76%	86.49%	81.08%	82.43%	82.43%	81.76%	81.08%	82.43%	89.19%	91.89%
Asad-Sarwar	87.84%	91.89%	91.22%	92.57%	89.86%	85.81%	89.86%	86.49%	89.19%	87.16%	90.54%	93.24%
Asad-Yousif Ali	85.14%	87.84%	85.14%	91.89%	86.49%	88.51%	86.49%	89.19%	86.49%	87.84%	90.54%	92.57%
Daryabadi-Maududi	80.41%	86.49%	81.08%	85.81%	74.32%	82.43%	74.32%	81.76%	77.03%	83.78%	86.49%	88.51%
Daryabadi-Pickthall	53.38%	55.41%	54.05%	54.05%	52.70%	64.19%	52.70%	64.19%	50.00%	62.84%	58.78%	60.14%
Daryabadi-Raza	83.78%	83.11%	83.78%	85.14%	87.16%	75.68%	75.00%	85.14%	84.46%	89.19%	90.54%	89.86%
Daryabadi-Sarwar	66.89%	72.97%	66.89%	70.27%	66.89%	77.03%	65.54%	75.00%	68.92%	75.68%	80.41%	77.70%
Daryabadi-Yousif Ali	89.86%	91.22%	89.86%	91.22%	88.51%	93.92%	88.51%	93.92%	87.84%	92.57%	91.89%	93.92%
Maududi-Pickthall	72.97%	85.14%	75.00%	83.78%	81.08%	80.41%	84.46%	79.73%	82.43%	79.05%	83.78%	85.14%
Maududi-Raza	57.43%	62.84%	57.43%	62.84%	65.54%	64.19%	65.54%	62.84%	64.86%	67.57%	70.95%	76.35%
Maududi-Sarwar	60.81%	67.57%	64.19%	66.89%	54.73%	61.49%	55.41%	63.51%	53.38%	63.51%	62.84%	72.30%
Maududi-Yousif Ali	52.70%	55.41%	57.43%	56.08%	59.46%	63.51%	59.46%	61.49%	64.86%	59.46%	73.65%	59.46%
Pickthall-Raza	81.76%	82.43%	80.41%	81.76%	79.05%	77.70%	80.41%	78.38%	79.05%	81.76%	87.84%	90.54%
Pickthall-Sarwar	63.51%	66.22%	64.86%	64.86%	58.78%	65.54%	58.78%	65.54%	60.81%	64.19%	70.27%	72.97%
Pickthall-Yousif Ali	87.84%	89.86%	87.84%	89.86%	83.11%	87.84%	83.11%	87.16%	82.43%	86.49%	93.24%	93.24%
Raza-Sarwar	63.51%	62.16%	64.19%	64.19%	65.54%	64.19%	66.22%	63.51%	66.89%	62.84%	75%	81.76%
Raza-Yousif Ali	64.86%	71.62%	65.54%	70.27%	64.86%	72.97%	64.86%	73.65%	66.22%	75.68%	77.03%	77.70%
Sarwar-Yousif Ali	78.38%	72.97%	78.38%	74.32%	68.24%	76.35%	68.24%	75.00%	70.27%	77.03%	79.73%	79.73%
Average	75.97%	78.67%	76.77%	78.96%	75.29%	78.28%	75.00%	78.47%	76.00%	79.02%	82.72%	83.98%
STD	0.1371	0.1288	0.1311	0.1337	0.1289	0.1105	0.1275	0.1128	0.1286	0.1142	0.1063	0.1090
Classifiers with accuracy >66.67%	14/21	16/21	14/21	16/21	14/21	15/21	13/21	15/21	15/21	16/21	19/21	19/21
*P*(*T* ≤ *t*) one-tail *H*0: *μ*(*VR*) ≤ *μ*(*M*3) *H*1: *μ*(*VR*)>*μ*(*M*3)											1.65E-05	2.95E-05

Similarly, the accuracy of the vocabulary richness features increased when ranking was applied to the features. These categories of features resulted in 82.72% using C4.5 and 83.98% using SVM. Even though one tail paired t-test showed that vocabulary richness outperformed network motifs when tested with the first dataset, network motifs outperformed vocabulary richness significantly with the second dataset as shown in Table 12.

**Table 12:** Classification results for applying ranking to motifs of size three and vocabulary richness as translator stylometry features for the 2nd corpus.

Features		Motifs Size Three with Nodes and Edges		Vocabulary Richness		Motifs and Vocabulary Richness	
Data Set	Translators	C4.5	SVM	C4.5	SVM	C4.5	SVM
First Part (52 Chapters)	Jarvis- Shelton	100%	100%	99.04%	98.08%	99.04%	100%
	Jarvis- Ormsby	87.50%	83.65%	82.69%	84.62%	87.50%	86.54%
	Ormsby- Shelton	100%	100%	98.08%	98.08%	100%	100%
Second Part (74 Chapters)	Jarvis- Shelton	99.32%	99.32%	94.59%	93.24%	99.32%	98.65%
	Jarvis- Ormsby	85.14%	87.16%	75.68%	75.68%	84.46%	90.54%
	Ormsby- Shelton	98.65%	98.65%	95.95%	94.59%	98.65%	97.97%
Average		95.10%	94.80%	91.00%	90.71%	94.83%	95.62%
STD		6.86%	7.37%	9.55%	8.87%	6.94%	5.68%
*P*(*T* ≤ *t*) one-tail *H*0: *μ*(*M*3) ≤ *μ*(*VR*) *H*1: *μ*(*M*3)>*μ*(*VR*)				0.0107	0.0343		

It was interesting to investigate the change in the performance of the two measures from the first dataset to the second dataset. The main difference between the two datasets is in the size of the chapters. In the first dataset, some chapters were as short as 33 words, while with the second dataset, the smallest chapter was 759 words. Network motifs showed better performance with the second dataset.

Additionally, results of the second dataset for both network motifs and vocabulary richness were better with average accuracy ranging from 90.71% to 95.62% as shown in Table 12. This enhancement in accuracy can be contributed to two factors: the first is the change in text size. The lengthy chapters may have allowed for syntactic variations to surface as good discriminators. The second factor is the special challenging characteristics of the Holy Qur’an as a religious book that made the translators keen to be close to the original text as much as possible and minimised their own bias and limited their space of freedom while translating.

The accuracy ranges shown in Tables 11 and 12 are sufficient to conclude that translators do have styles of their own. These results provided an answer to our first question on the existence of translators’individual styles. Our results provided evidence that translators can be identified through individual styles. The use of network motifs in this research can be seen as capturing patterns on the syntactic level.

In summary, this research has contributed significantly to the study of translator stylometry. Through a series of experiments this research divulged the significance of network motifs as a criterion for measuring translator stylometry. Network motifs showed higher performance levels compared to traditional stylistic features such as vocabulary richness. Additionally, if we compare the performance of the network motifs feature group to a random chance of equal opportunity distribution, its significance as a new feature can be seen clearly. A random chance of two class distribution is 50%, while network motifs achieved an average accuracy of 79%.

Further analysis of the three-class classification problem and the effect of applying feature selection is presented in S2 Appendix. In the case of a three-class classification problem, a classifier based on random guessing would have a classification accuracy of 33% on average. We can see that network motifs achieved 68.24% without feature selection, and 81.08% after applying feature selection. Additionally, network motifs have outperformed vocabulary richness when feature selection has been applied as presented in S2 Appendix. A time analysis has been conducted and added to S1 Appendix to compare the time required for extracting both local and global network features.

A summary of the findings on selecting the best method for translator stylometry identification is presented below:

- The problem of stylometry identification is a challenging problem that classic computational linguistic tools failed to identify.
- Non-Parametric Scaling (For example the ranking method used in this study) provides a useful tool that enabled the usage of classic features as vocabulary richness, that initially failed to identify translators. The classification accuracy of a decision tree C4.5 that uses vocabulary richness increased from 53.59% to 91% for the second corpus after using the non-parametric scaling method discussed earlier as shown in Tables 10 and 12.
- Without the use of non-parametric scaling, an SVM classifier that uses network motifs was able to identify translators with accuracy of 77.14% for the 2nd corpus compared to 57% for the vocabulary richness as shown in Table 10.
- Using non-parametric scaling, network motifs again outperformed vocabulary richness with the second corpus. The main difference between the two datasets was in the number of words per chapter. The first corpus has very short text with 33 words per chapter on average, while the shortest text in the second corpus is 797 words per chapter. This suggests that network motifs are better used as a stylistic feature indicator for moderate and large size texts, unless they are accompanied with feature selection as we discuss below.
- By evaluating motifs of size three versus size four, with and without adding the network size indicators parameters, best results were obtained using all motifs of size three and four in addition to the number of nodes and edges in the network as shown in Table 11.
- Considering the analysis of applying feature selection proposed in S2 Appendix, feature selection combined by non-parametric scaling supplied network motifs achieved the balance needed to perform with an accuracy of 81.08% for the three-class classification task with the first corpus compared to 34.19% with no ranking and no feature selection, and 68.24% with raking alone. Therefore, it is recommended that the feature selection method is applied, accompanied by the non-parametric scaling for the translator stylometry identification task.

## Conclusion

In this paper, we addressed the challenging problem of translator stylometry, which received limited research attention. We demonstrated that vocabulary richness features can be used to detect translator stylometry, contrary to the claims made by Mikhailov and Villikka [10]. Detecting network motifs in a network can mimic detecting translators’repeated patterns in their writing. Although using network motifs as a stylistic feature failed to identify translators, representing the data using ranking to express the relationship between different usages of the same pattern in comparison to different translators introduced promising results. It provided data transformation that allowed minimized the effect of the original text on the analysis. Some of the generated classifiers achieved accuracy of 97.97%, while the overall average of accuracy reached 79.02% for the case of two translators for the Holy Qur’an corpus. Applying feature selection with the proposed approach achieved an accuracy of 81.08% in the case of three classes (translators) problem on the same dataset. Additionally, network motifs outperformed vocabulary richness as a stylometric feature for the second dataset investigated in this study, which is a Spanish novel, with an average accuracy of 95.10% that reached 100% for a number of cases.

The first contribution in this paper is in providing evidence for the existence of translator stylometry using classic features and network-based features. The second contribution is the effectiveness of network motifs as a new method in detecting translator stylometry. Both of these contributions encourage further studies in translator stylometry identification. Extending this analysis to a larger number of books and translators is recommended in future research studies.

## Supporting information

S1 Appendix Time analysis. (PDF)

S2 Appendix Evaluation of multi-class classification and feature selection for translator stylometry identification using network motifs features. (PDF)
