# Supplementary material for: Network motifs for translator stylometry identification
Source: PLoS One. 2019 Feb 8;14(2):e0211809. doi: 10.1371/journal.pone.0211809 (PMC6368295; doi:10.1371/journal.pone.0211809)
Supplement: S1 Appendix — (PDF) [file pone.0211809.s001.pdf]

S1 Appendix: Time Analysis

We used the 2nd Corpus: Spanish-to-English Translations of “Don Quixote” for the time analysis conducted in the appendix.

Before analysing the time required for network feature extraction, we analysed the relationship between text size and the corresponding network size. Network size is different from text size as nodes represent unique words only. This relationship can be seen in Figure 1(a).

For this analysis, we used translator “Shelton” corpus as a sample. The number of words in Shelton’s corpus ranged from 1820 words to 8921 words. The size of the generated networks ranged from 599 to 1637 nodes. Calculating average degree reflects an important network property. The effect of network size on network complexity has been investigated in Figure 1(b) via the relationship between the number of words in each chapter and the average degree of the corresponding network. Apparently, there is no definite relationship between the text size and average degree of the networks.

The effect of sample text size on the time required to extract both network motifs and to compute network global features is shown in Figures 1(c) and 1(d). Network motifs extraction is more time consuming than computing network global features, but both of them show similar

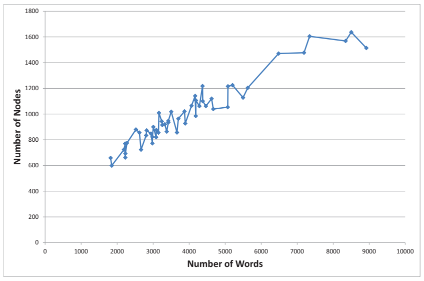

(a) Relation between Text Size and Network Size

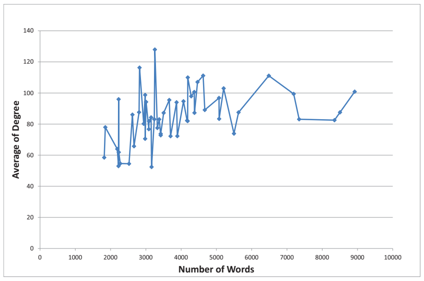

(b) Relation between Text Size and Average of Degree of the Network

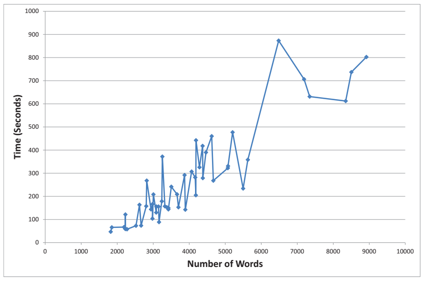

(c) Relation between Text Size and Time Required to Count Network Motifs

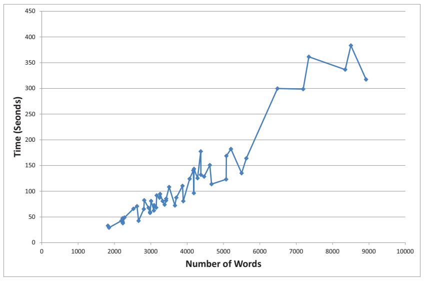

(d) Relation between Text Size and Time Required to compute Global Features

Figure 1: Effect of Text size on the Computational Requirements in the 52 Chapters of the First Part of *Don Quixote* by Translator Shelton

trend in their dependence on text size and the follow-up effect as measured by the computational time. The time required for network motifs extraction ranged from 47 seconds to 873 seconds (14.55 minutes), while the time required for global features calculation ranged from 29 seconds to 383 seconds(6.38 minutes).
