# Supplementary material for: Network motifs for translator stylometry identification
Source: PLoS One. 2019 Feb 8;14(2):e0211809. doi: 10.1371/journal.pone.0211809 (PMC6368295; doi:10.1371/journal.pone.0211809)
Supplement: S2 Appendix — (PDF) [file pone.0211809.s002.pdf]

S2 Appendix: Evaluation of Multi-class Classification and Feature Selection for Translator Stylometry Identification Using Network Motifs Features

In this section, we describe an experiment which was conducted to evaluate the performance of network motifs features for translator stylometry identification in the case of multiple translators involved in the classification. This experiment included three-class classification for five translators.

For this experiment, we applied the ranking method, described in Method III using the 214 features that were described in the same section which are: all motifs of size three and size four in addition to the number of nodes and edges of each generated network. Then, we applied feature selection technique using **wrapper algorithm** as the attribute evaluator with C4.5 classifier (WEKA implementation) with 10 folds cross-validation. The applied search method for the feature selection was the greedy hill climbing **best first** algorithm.

Table 1 reveals the results of three class classification using three group of features. The first group includes all network motifs related features, the second group includes vocabulary richness features, and the third group combines the two sets of features together. The average accuracy achieved using network motifs as translator stylometry identification features before applying the ranking method was 34.19%, which was raised into 68.24% after applying the ranking method for all possible combinations of the three classes classification problem.

Using vocabulary richness for the same problem showed slightly better accuracy with average of 42.03%, which was enhanced significantly to 77.52% after applying the ranking method. Combining the two group of features didn't enhance the accuracy of classification. Additionally, although vocabulary richness features performed better than network motifs, network motifs still performed well in terms of being able to classify three classes problem with average accuracy of 68.24% without feature selection. A random probability of correct classification in the case of equal opportunities for three classes problem is 33.33%. Thus, network motifs can be recommended as a feature group that can capture some stylistic signatures.

Applying feature selection for the same feature sets enhanced the average accuracy as shown in Table 2. Network motifs achieved 81.08%, and vocabulary richness achieved 80.41%. This time, combining the two group of features before applying feature selection enhanced the average accuracy up to 83.20%. It can be observed that network motifs outperformed vocabulary richness after applying feature selection. Different motifs were selected independently for each different combination of translators. The implication of that is that the feature sets used for classification differ as the pairs of translators change.

Another observation that can be made based on Table 2 is that except from the 2nd, 3rd, and 7th combination of translators, vocabulary richness features were selected in each combination of translators. However, network motifs were always part of the selected features for each combination of translators.

Table 1: Results of three classes classification for the five translators (Asad, Daryabadi, Pickthall , Raza, YousifAli) using C4.5 without Feature Selection

Data Presentation	No ranking			Ranking		
Feature group	Network motifs	VR	VR + Network Motifs	Network motifs	VR	VR + Network Motifs
Translators	Accuracy	Accuracy	Accuracy	Accuracy	Accuracy	Accuracy
Asad-Daryabadi-Pickthall	33.78%	46.40%	44.14%	54.96%	68.47%	59.91%
Asad-Daryabadi-Raza	37.39%	46.40%	43.69%	75.23%	83.33%	81.53%
Asad-Daryabadi-YousifAli	37.39%	46.85%	48.65%	81.53%	87.84%	88.29%
Asad-Raza-Pickthall	35.59%	50.90%	50.90%	75.23%	86.49%	78.38%
Asad-Raza-YousifAli	32.43%	54.50%	45.50%	71.62%	77.03%	72.07%
Asad-YousifAli-Pickthall	36.04%	47.75%	51.35%	82.43%	88.29%	87.39%
Daryabadi-Pickthall-YousifAli	31.98%	30.18%	36.04%	52.70%	67.57%	61.71%
Daryabadi-Raza-Pickthall	31.98%	32.88%	31.98%	55.86%	61.71%	58.11%
Daryabadi-Raza-YousifAli	32.88%	30.18%	34.23%	69.37%	76.58%	72.52%
Raza-Pickthall-YousifAli	32.43%	34.23%	29.28%	63.51%	77.93%	76.13%
Average Accuracy	34.19%	42.03%	41.58%	68.24%	77.52%	73.60%
STD	0.022	0.092	0.081	0.110	0.092	0.109

Table 2: Results of three classes classification for the five translators (Asad, Daryabadi, Pickthall , Raza, YousifAli) using C4.5 with Feature Selection

Feature group	Network motifs		VR		VR + Network Motifs	
Translators	Accuracy	Selected Features	Accuracy	Selected Features	Accuracy	Selected Features
Asad-Daryabadi-Pickthall	78.38%	M4_ID16, M4_ID50, M4_ID63	75.23%	V,K	72.07%	M4_ID16, M4_ID50, M4_ID63, M4_ID146, N
Asad-Daryabadi-Raza	86.04%	M3_ID2, M3_ID7, M4_ID49, M4_ID97, M4_ID123	87.39%	N,Vk,K	86.04%	M3_ID2, M3_ID7, M4_ID49, M4_ID97, M4_ID123
Asad-Daryabadi-YousifAli	90.09%	M4_ID8, M4_ID33, M4_ID70, M4_ID105	88.74%	V,W	90.09%	M4_ID8, M4_ID33, M4_ID70, M4_ID105
Asad-Raza-Pickthall	84.23%	M4_ID11, M4_ID32, M4_ID65, M4_ID85, M4_ID159, M4_ID180, M4_ID182	87.39%	N,V,K,W	88.74%	M4_ID18, M4_ID19, M4_ID113, N
Asad-Raza-YousifAli	84.68%	M3_ID2, M4_ID62 , M4_ID108, no_nodes	78.38%	V,W	87.84%	M4_ID30, M4_ID66, M4_ID108, V
Asad-YousifAli-Pickthall	88.74%	M4_ID74, M4_ID103, M4_ID113, M4_ID181, no_edges	90.54%	N,V	94.59%	M3_ID1, M4_ID30, M4_ID53, M4_ID74, M4_ID83, M4_ID108, M4_ID166, M4_ID178, N, V
Daryabadi-Pickthall-YousifAli	96.37%	M3_ID4, M4_ID60, M4_ID94, M4_ID110, M4_ID116, M4_ID123, M4_ID177, no_edges	68.92%	N,K	69.37%	M3_ID4, M4_ID60, M4_ID94, M4_ID110, M4_ID116, M4_ID123, M4_ID177, no_edges
Daryabadi-Raza-Pickthall	64.86%	M3_ID9, M3_ID12, M4_ID43, M4_ID51, M4_ID121, M4_ID129, M4_ID192, no_edges	67.57%	N,K	73.87%	M3_ID9, M3_ID12, M4_ID28, M4_ID48, M4_ID54, M4_ID60, M4_ID83, M4_ID122, M4_ID129, M4_ID189, M4_ID196, no_edges, N
Daryabadi-Raza-YousifAli	82.88%	M3_ID2, M4_ID5, M4_ID8, M4_ID68, M4_ID75, M4_ID79, M4_ID86, M4_ID98, M4_ID132, M4_ID143, no_nodes, no_edges	78.38%	N,V,R	83.33%	M4_ID32, M4_ID79, M4_ID145, no_nodes, V
Raza-Pickthall-YousifAli	81.53%	M4_ID63, M4_ID76, M4_ID86, no_nodes, no_edges	81.53%	N,V	86.04%	M4_ID5, M4_ID24, M4_ID93, no_nodes, N, V
Average	81.08%		80.41%		83.20%	
STD	0.082		0.082		0.085	
